# Bronchial tree of the human embryo: Categorization of the branching mode as monopodial and dipodial

**DOI:** 10.1371/journal.pone.0245558

**Published:** 2021-01-15

**Authors:** Sena Fujii, Taiga Muranaka, Jun Matsubayashi, Shigehito Yamada, Akio Yoneyama, Tetsuya Takakuwa

**Affiliations:** 1 Human Health Science, Graduate School of Medicine, Kyoto University, Kyoto, Japan; 2 Congenital Anomaly Research Center, Graduate School of Medicine, Kyoto University, Kyoto, Japan; 3 SAGA Light Source, Saga, Japan; Leibniz Institute on Aging - Fritz Lipmann Institute (FLI), GERMANY

## Abstract

Some human organs are composed of bifurcated structures. Two simple branching modes—monopodial and dipodial—have been proposed. With monopodial branching, child branches extend from the sidewall of the parent branch. With dipodial branching, the tip of the bronchus bifurcates. However, the branching modes of the human bronchial tree have not been elucidated precisely. A total of 48 samples between Carnegie stage (CS) 15 and CS23 belonging to the Kyoto Collection were used to acquire imaging data with phase-contrast X-ray computed tomography. Bronchial trees of all samples were three-dimensionally reconstructed from the image data. We analyzed the lobar bronchus, segmental bronchus, and subsegmental bronchus. After calculating each bronchus length, we categorized the branching mode of the analyzed bronchi based on whether the parent bronchus was divided after generation of the analyzed bronchi. All lobar bronchi were formed with monopodial branching. Twenty-five bifurcations were analyzed to categorize the branching mode of the segmental and subsegmental bronchi; 22 bifurcations were categorized as monopodial branching, two bifurcations were not categorized as any branching pattern, and the only lingular bronchus that bifurcated from the left superior lobar bronchus was categorized as dipodial branching. The left superior lobar bronchus did not shorten during the period from CS17 or CS18, when the child branch was generated, to CS23. All analyzed bronchi that could be categorized, except for one, were categorized as monopodial branching. The branching modes of the lobar bronchus and segmental bronchus were similar in the mouse lung and human lung; however, the modes of the subsegmental bronchi were different. Furthermore, remodeling, such as shrinkage of the bronchus, was not observed during the analysis period. Our three-dimensional reconstructions allowed precise calculation of the bronchus length, thereby improving the knowledge of branching morphogenesis in the human embryonic lung.

## Introduction

Many organs, including the lungs and kidneys, have complicated structures resulting from a series of bifurcations. An understanding of branching morphogenesis is essential to the diagnosis and treatment of congenital anomalies. However, this morphogenesis process is not well-known. Previous studies have proposed various types of branching mode categorizations to describe these bifurcations. Palmer [1] introduced the following three branching modes for the human lung: lateral budding, dichotomous branching, and trichotomous branching. Metzger et al. [2] proposed the following three patterns for mouse bronchi: domain branching, planar bifurcation, and orthogonal bifurcation. Real-time analyses of mouse kidneys revealed the following three main branching modes: terminal bifid branching, terminal trifid branching, and lateral branching [3]. Despite differences among the analyzed species or organs, these studies showed that two simple and essential branching modes, monopodial branching and dipodial branching, are common among all these species. Child branches (CBr) are generated at the sidewall of the parent branch (PBr) with monopodial branching, whereas the tip of the bronchus is bifurcated with dipodial branching. The former mode corresponds to domain branching, and the latter mode corresponds to planar and orthogonal bifurcation [2].

Detailed morphological changes in the human bronchial tree during the embryonic period were observed in our recent study [4]. We noticed the following two characteristics of the human embryonic bronchial tree: the lobar bronchi appeared to be formed monopodially [5], and the human embryonic bronchial tree seemed to have a similar structure until the subsegmental bronchus. In particular, the trachea and lobar bronchi showed no individual differences. The structures of the segmental and subsegmental bronchi in each sample showed individual variability. Although 14 variations were found at the segmental level [4], these variations have been reported by adult lung studies, and the quantity of these variations was few compared to those studies [6–10]. Additionally, the length of the bronchus forming the bronchi in children did not change dramatically during development, in other words, the bronchi did not shrink suddenly during the embryonic period. Therefore, the branching modes can be categorized as monopodial and dipodial by measuring the bronchus length.

The present study aimed to analyze how the proximal bronchus, lobar bronchus, segmental bronchus, and subsegmental bronchus of the human lung branch off during the embryonic period. We reconstructed three-dimensional overall branching trees of samples using phase-contrast X-ray computed tomography images and categorized them as monopodial branching and dipodial branching using the bronchus length.

## Materials and methods

### Human embryo specimens

Approximately 44,000 human embryos comprising the Kyoto Collection are stored at the Congenital Anomaly Research Center of Kyoto University [11, 12]. In most cases, the pregnancies were terminated during the first trimester for socioeconomic reasons under the Maternity Protection Law of Japan. The samples were collected from 1963 to 1995 according to the regulations pertaining to each time period. For instance, written informed consent was not required from parents at that time. Instead, parents provided verbal informed consent to have these specimens deposited, and each participant’s consent was documented in the medical record. All samples were anonymized and de-identified. The ethics committee of the Kyoto University Faculty and Graduate School of Medicine approved this study, which used human embryo and fetal specimens (E986, R0316). Aborted embryos brought to the laboratory were measured, examined, and staged using the criteria of O’Rahilly and Müller [13]. Whole embryonic samples were fixed with 10% formalin. A total of 48 human embryos between Carnegie stage (CS) 15 and CS23 were selected from the Kyoto Collection (n = 7 each at CS15, CS16, CS17, CS18, and CS19; n = 4 each at CS20 and 21; n = 3 at CS22; n = 2 at CS23). All samples were free of overt damage and anomalies. The lungs were not inflated for imaging. These samples were also used in our previous study [4].

### Image acquisition and three-dimensional reconstruction

**Fig 1:**
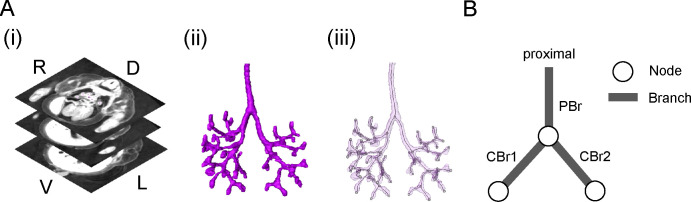
Image processing of the bronchial tree and definitions of the node and branch. (A) Image acquisition and three-dimensional reconstruction were performed. (i) Serial transverse section using phase-contrast X-ray computed tomography (ID 18071). D, dorsal; L, left; R, right; V, ventral. (ii) Reconstructed bronchial tree. (iii) Transparent reconstruction and centerline tree. (B) An illustration of bifurcation indicating the definitions of node and branch in the present study. Simplified centerline indicating bifurcation. The white and black lines represent the node and branch, respectively. The proximal branch of the bifurcation was defined as the parental branch (PBr), and peripheral branches were defined as child branches (CBr).

The three-dimensional phase-contrast X-ray computed tomography image acquisition conditions have been described previously [14]. Briefly, specimens were visualized with a phase-contrast imaging system fitted with a crystal X-ray interferometer. The system was set up at the vertical wiggler beam line (PF BL14C; Photon Factory, Tsukuba, Japan). Phase-contrast X-ray computed tomography data from selected embryos were analyzed precisely as serial two-dimensional and reconstructed three-dimensional images. The structure of the bronchial tree was reconstructed for all samples using Amira software version 6.2.0 (Visage Imaging GmbH, Berlin, Germany) (Fig 1A). The center of the airway was observed linearly with the centerline module; then, it was manually corrected by plotting the bifurcation point at the base and tip of the swellings. During this study, the terms “node” and “branch” were defined (Fig 1B). The node was either the point at which bifurcation occurred or the terminal point. The branch was the trunk of the bronchus bounded by two nodes. An analyzed bifurcation was composed of the PBr and CBr.

### Developmental phase of the bronchial tree during lobar bronchus formation

The following three developmental phases of the embryonic bronchial tree during lobar bronchus formation were determined based on morphological features [4]. During phase 1, the primary bronchus had no lobar swelling. The primary bronchus formed an almost symmetrical Y shape. During phase 2, the bronchus had lobar swellings that emerged from the middle of each bud. These swellings were at the right middle lobar bronchus (RMLB) and left superior lobar bronchus (LSLB). The bronchial trees still exhibited almost total symmetry. During phase 3, the right superior lobar bronchus (RSLB) branched off. The bronchus had all five distinct lobar swellings. The right and left primary bronchi showed characteristic asymmetry. All 14 samples at CS15 and CS16 were classified as any of these three phases.

### Categorization of the branching mode based on length

**Fig 2:**
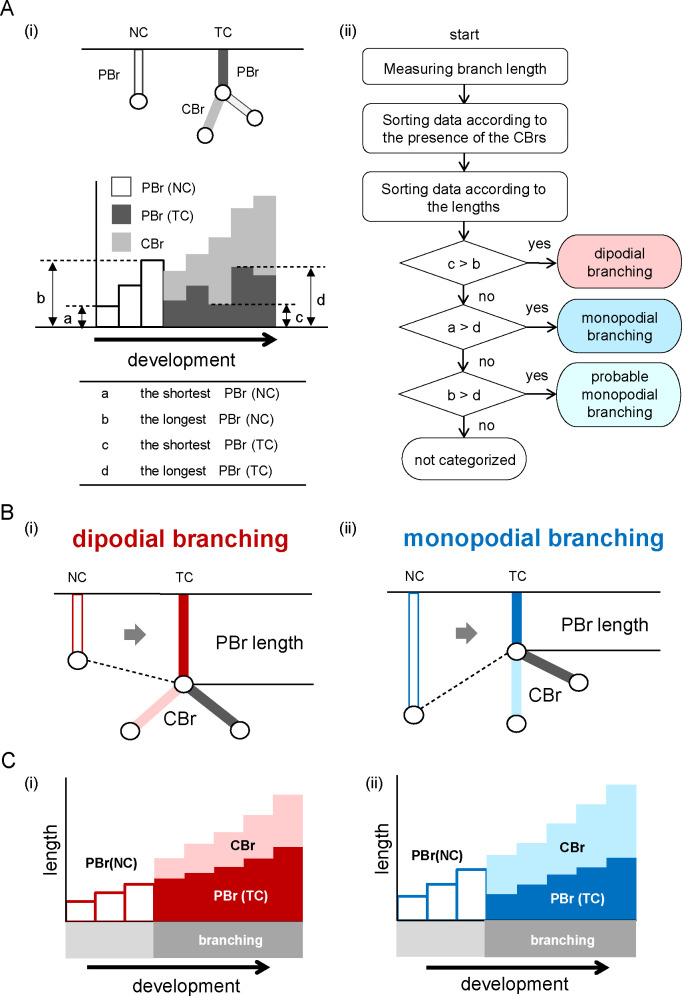
Categorization flowchart and schematic diagram indicating monopodial and dipodial branching. (A) (i) Illustration showing the definition of NC and TC (upper), and the definitions of a, b, c, and d (lower). Here, a and b are the shortest and longest PBr(NC) lengths, respectively, and c and d are the shortest and longest PBr(TC) lengths, respectively. CBr, child branch; NC, bifurcation that generates no child branches; PBr, parent branch; TC, bifurcation that generates two child branches. (ii) Flowchart of the branching mode categorization. (B) Illustration showing the change in PBr length with dipodial and monopodial branching. The PBr length may not shrink or elongate with dipodial branching (i), but may shrink with monopodial branching (ii) just after generation of CBr. (C) Schematic graphs of PBr length subjected to bifurcation by dipodial and monopodial branching. The branching mode was categorized as dipodial or monopodial branching according to the change in the PBr length. When the PBr(TC) length remained constant with the birth of CBr, the CBr were formed with dipodial branching (i). When the PBr(TC) length was shortened with the birth of CBr, the CBr were formed with monopodial branching (ii).

The branch length and presence of CBr were deemed to reflect the degree of development in the present study. Therefore, for categorization of the branching mode, we plotted a graph wherein branch lengths were arranged according to the size and presence of CBr (Fig 2A, i). The categorization process is explained in a flowchart (Fig 2A, ii). We measured the PBr length (and CBr length if generated already) of the analyzed bifurcation of all individual samples. The length of each branch was calculated using MATLAB (version R2018a; MathWorks, Natick, MA, USA) algorithms based on the orthogonal coordinates of the voxels of each reference point. Data from all samples were grouped into a no-child group (NC) and a two-child group (TC); the bifurcations that generated no CBr and two CBr were categorized into the NC and TC groups, respectively. Data were excluded when the PBr of the analyzed bifurcation was absent and the CBr generated further descendant branches. Subsequently, in the NC group, data were sorted according to the length of the PBr [PBr(NC)], while in the TC group, they were sorted according to the total length of the PBr [PBr(TC)] and CBr. Lastly, NC and TC graphs were merged (Fig 2A, i).

Branching mode was categorized into monopodial and dipodial branching based on whether the length of the PBr was divided after the generation of the CBr. The shortest and longest PBr lengths were defined; furthermore, the shortest and longest PBr(NC) lengths (Fig 2A, i, a and b) and the shortest and longest PBr(TC) lengths (Fig 2A, i, c and d) were observed. The branching mode of an analyzed bifurcation was categorized as dipodial branching when the shortest PBr(TC) length was longer than the longest PBr(NC) length (c>b; Fig 2B, i and 2C, i). When the shortest PBr(NC) length was longer than the longest PBr(TC) length (a>d; Fig 2B, ii and 2C, ii), the mode was defined as monopodial branching. Finally, the mode was presumed to be probable monopodial branching when the longest PBr(TC) length was longer than the longest PBr(NC) length (b>d; Fig 2A, ii). When the analyzed bifurcation did not apply to any of these, the pattern could not be categorized.

## Results

### Branching mode of the lobar bronchi

**Fig 3:**
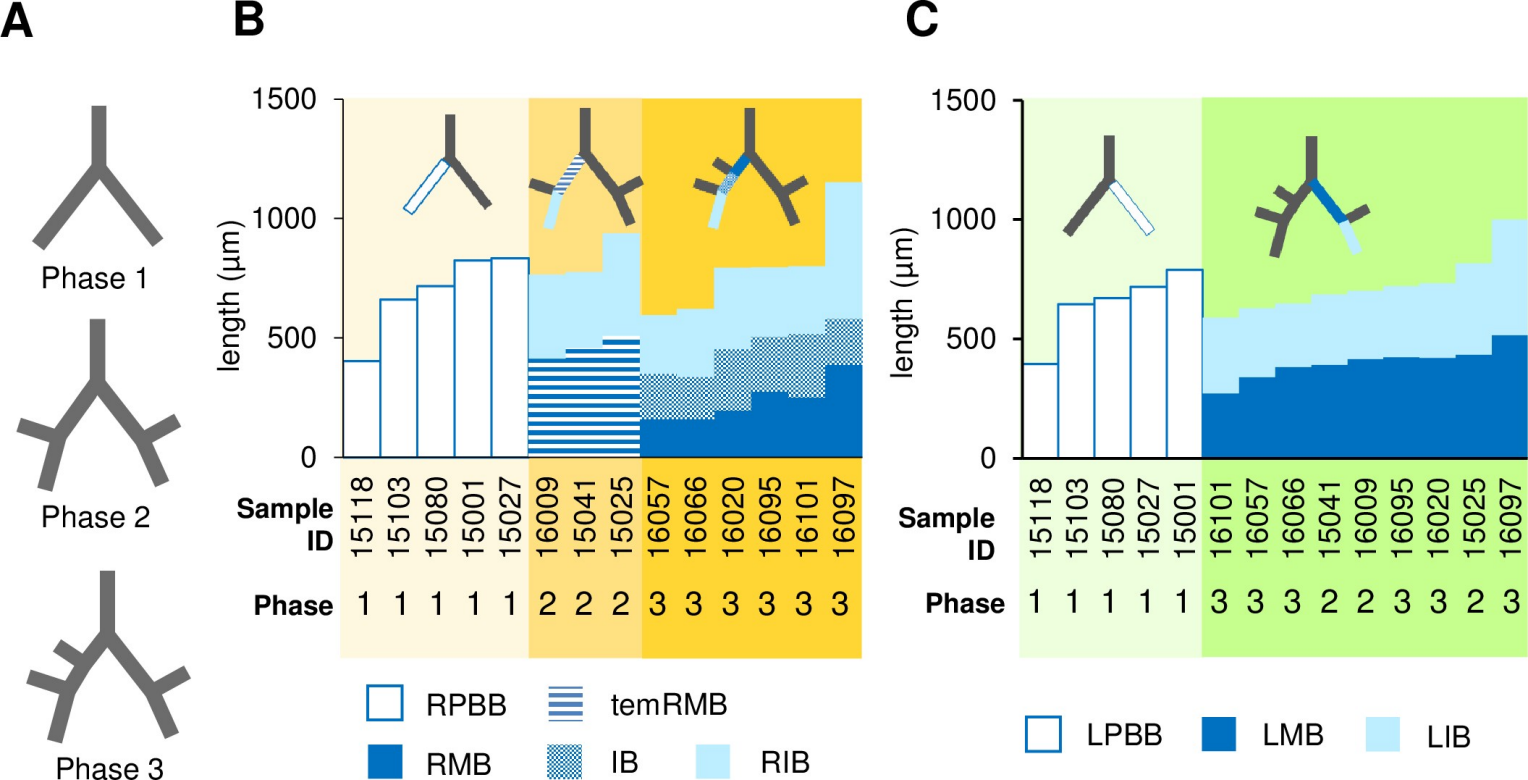
Branching mode of the lobar bronchi. (A) Idealized diagram of the generation period of the lobar bronchus. No lobar bronchus existed during phase 1. The RMLB and LSLB sprouted during phase 2. During phase 3, all lobar bronchi were formed. The length changes of the right proximal bronchi (B) and the left proximal bronchi (C) are shown. Compared with the RPBB length during phase 1, the RMB length and total length of RMB and IB were shorter (B). Similarly, the LMB length was shorter than the LPBB length (C). IB, intermediate bronchus; LIB, left inferior bronchus; LMB, left main bronchus; LPBB, left primary bronchial bud; RIB, right inferior bronchus; RMB, right main bronchus; RPBB, right primary bronchial bud; temRMB, temporary RMB branch from the tracheal bifurcation to the base of the right middle lobe.

To categorize the branching mode of the lobar bronchus, we analyzed the samples during phases 1 and 3. By comparing the PBr length before and after CBr generation, our results demonstrated that lobar bronchi were formed with the monopodial branching mode. Monopodial branching comprised one (RSLB) bifurcation and probable monopodial branching comprised two bifurcations (RMLB and LSLB) (Fig 3 and Table 1).

**Table 1:** Categorization of the branching mode of the lobar bronchus.

Bifurcation	PBr(NC)/PBr(TC)/CBr	PBr(NC) (μm)			PBr(TC) (μm)			Categorization
		a	-	b	c	-	d	
RMLB	RPBB/temRMB/RIB	404	-	833	414	-	509	mono*
RSLB	temRMB/RMB/IB	414	-	509	161	-	389	mono
LSLB	LPBB/LMB/LIB	396	-	789	273	-	516	mono*

The first row indicates the analyzed bifurcation. The second row indicates bronchi that were used for categorization of the analyzed bronchus. CBr, child branch; IB, intermediate bronchus; LIB, left inferior bronchus; LMB, left main bronchus; LPBB, left primary bronchial bud; LSLB, left superior lobar bronchus; mono, monopodial branching; mono*, probable monopodial branching; PBr(NC), parent branch of bifurcation that generates no child branches; PBr(TC), parent branch of bifurcation that generates two child branches; RIB, right inferior bronchus; RMB, right main bronchus; RMLB, right middle lobar bronchus; RPBB, right primary bronchial bud; RSLB, right superior lobar bronchus; temRMB, temporary right main bronchus branch from the tracheal bifurcation to the base of the right middle lobe.

### Deducing the branching mode of the segmental and subsegmental bronchi

**Fig 4:**
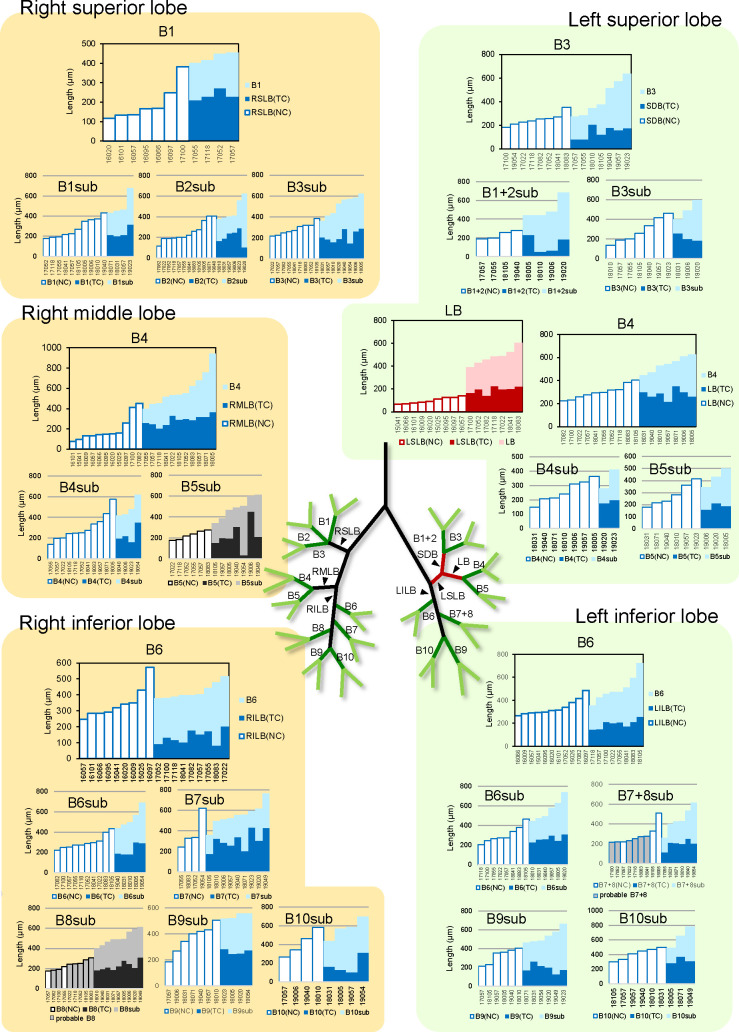
Branching mode categorizations of segmental and subsegmental bronchi. The length change of each segmental or subsegmental bronchus is shown. The graph titles indicate the bifurcation. X axis is sample number and Y axis is length. The graph color reflects the categorization of the bronchus. The red graph represents dipodial branching. The blue graph represents monopodial and probable monopodial branching. The graph of the uncategorized bronchus is monochrome. In the B8(7+8)sub graph, a bordered gray bar graph describes the probable B8(7+8) length, which was classified as B8(7+8) based on the orientation in immature samples. BXsub, subsegmental bronchus of BX; LB, lingular bronchus; LSLB, left superior lobar bronchus; RILB, right inferior lobar bronchus; RMLB, right middle lobar bronchus; RSLB, right middle lobar bronchus; RSLB, right superior lobar bronchus; SDB, superior division bronchus.

We analyzed 25 bifurcations. Selected samples for each bifurcation are shown in S1 Table. Two bifurcations were categorized as monopodial branching and 20 were categorized as probable monopodial branching; the only lingular bronchus that bifurcated from the LSLB was categorized as dipodial branching. The remaining two bifurcations were not categorized as any branching mode (Table 2 and Fig 4).

**Table 2:** Branching modes of segmental and subsegmental bronchi.

Bifurcation	PBr(NC and TC) / CBr	PBr(NC) (μm)			PBr(TC) (μm)			Categorization
		a	-	b	c	-	d	
B1	RSLB / B1	117	-	382	210	-	272	mono*
B1sub	B1 / B1sub	176	-	433	200	-	318	mono*
B2sub	B2 / B2sub	113	-	407	104	-	291	mono*
B3sub	B3 / B3sub	217	-	385	148	-	289	mono*
B4	RMLB / B4	75	-	451	206	-	367	mono*
B4sub	B4 / B4sub	139	-	574	162	-	352	mono*
B5sub	B5 / B5sub	175	-	276	37	-	450	not categorized
B6	RILB / B6	247	-	572	81	-	203	mono
B6sub	B6 / B6sub	221	-	432	182	-	299	mono*
B7sub	B7 / B7sub	240	-	621	177	-	433	mono*
B8sub	B8 / B8sub	175	-	218	186	-	310	not categorized
B9sub	B9 / B9sub	185	-	504	245	-	282	mono*
B10sub	B10 / B10sub	266	-	584	104	-	313	mono*
LB	LSLB / LB	67	-	139	141	-	226	di
B3	SDB / B3	184	-	353	84	-	209	mono*
B1+2sub	B1+2 / B1+2sub	190	-	278	54	-	231	mono*
B3sub	B3 / B3sub	136	-	461	185	-	255	mono*
B4	LB / B4	225	-	404	219	-	354	mono*
B4sub	B4 / B4sub	148	-	363	175	-	198	mono*
B5sub	B5 / B5sub	177	-	413	159	-	210	mono*
B6	LILB / B6	264	-	484	148	-	259	mono
B6sub	B6 / B6sub	205	-	464	228	-	309	mono*
B7+8sub	B7+8 / B7+8sub	219	-	509	112	-	253	mono*
B9sub	B9 / B9sub	210	-	405	128	-	265	mono*
B10sub	B10 / B10sub	301	-	500	285	-	371	mono*

The first row indicates the analyzed bifurcation. The second row indicates bronchi that were used for categorization of the analyzed bronchus. CBr, child branch; di, dipodial branching; LB, lingular bronchus; LILB, left inferior lobar bronchus; LSLB, left superior lobar bronchus; mono, monopodial branching; mono*, probable monopodial branching; NC, bifurcation that generate no child branches; PBr, parent branch; RILB, right inferior lobar bronchus; RMLB, right middle lobar bronchus; RSLB, right superior lobar bronchus; TC, bifurcation that generates two child branches.

**Fig 5:**
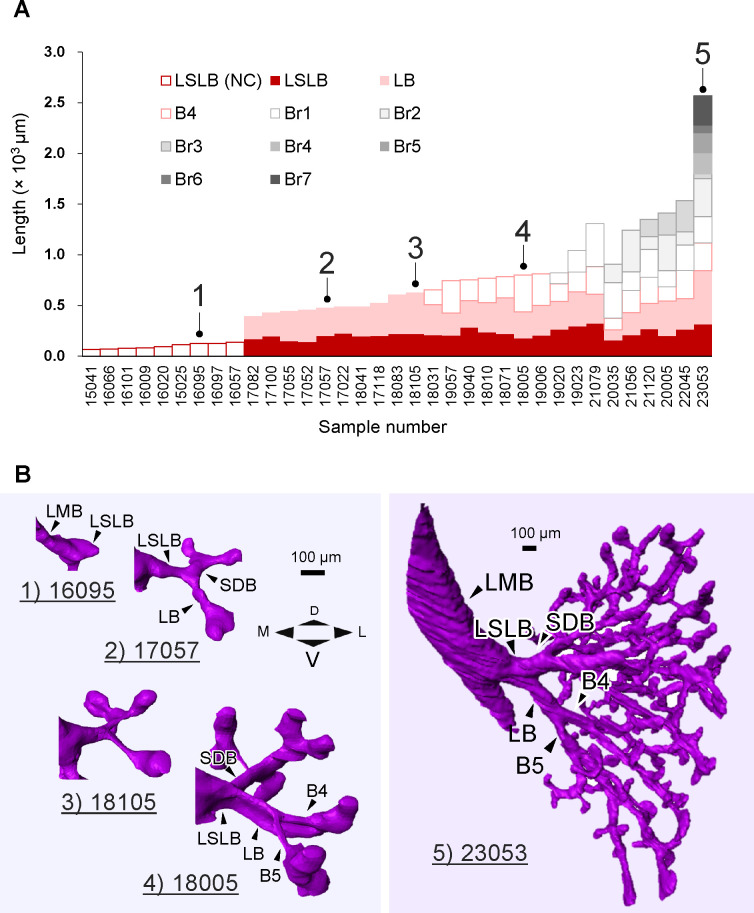
The length of the left superior lobar bronchus did not become shorter with growth. (A) The total length of the passage from the LSLB to the lateral peripheral bronchus. These lengths were sorted according to the generation of passage; subsequently, they were sorted in ascending order. Reconstructions of samples with numbers 1–5 are shown in (B). (B) Representative reconstructions indicated morphological changes according to growth. The LSLB length was almost constant. Scale bar: 100 μm. Ventrocranial view. BrX, branch without an identified anatomical term; D, dorsal; L, lateral; LB, lingular bronchus; LMB, left main bronchus; LSLB, left superior lobar bronchus; M, medial; NC, no child; SDB, superior division bronchus; V, ventral.

The superior division bronchus and LB were exceptions that formed with dipodial branching. Dipodial branching was only observed at this bifurcation. The length change of the LSLB itself and passage from the LSLB to the lateral peripheral bronchus are shown in Fig 5. The total length of the left superior lobe passage grew longer with development. The LSLB length after formation of CBr seemed to gradually increase and reached 324 μm (the sample with ID 21079). The shortest LSLB (141 μm for the sample with ID 17052) was approximately the same length as the longest LSLB(NC) (139 μm for the sample with ID 16057). Therefore, the peripheral branches repeatedly bifurcated, but the LSLB length did not shorten.

## Discussion

The two branching modes, monopodial and dipodial, are necessary to fill the whole lung space. If the bronchial tree was formed with only dipodial branching, then the end branches would be arranged at the edge [15]. Monopodial branching enables filling of the lung interior. A previous study demonstrated that monopodial branching generates a central structure and dipodial branching forms the edge and interior of the bronchial tree [2]. Therefore, categorization of the two branching modes will contribute to elucidating the morphogenesis of the human bronchial tree.

We categorized the branching mode of the bronchus based on whether the length of the parent bronchus was divided after the formation of the CBr. The precise calculations in the present study indicated that the lobar buds were given off from the side of the primary buds. Streeter reported that the lobar bronchus of the human embryonic lung sprouts monopodially during CS15 [5]. Although the results of the previous report of the branching mode of the lobar bronchus were estimated based on only visual assessments, our study statistically confirmed these findings using length measurements.

The present study revealed similarities and differences in the branching modes of the murine and human lung. Metzger demonstrated that the central bronchi, lobar bronchus, and segmental bronchus in mouse lungs arose through monopodial branching, which is called domain branching [2]. Similarly, our data revealed that lobar and segmental bronchi were formed with monopodial branching in the human lungs. The current study indicates that the lobar and segmental bronchi seem to be formed with a similar pattern (monopodial branching) in mice and humans. However, the branching mode of the subsegmental bronchus showed a difference. Metzger et al. [2] suggested that the subsegmental bronchus, which is the second-generation bronchus of the lobar bronchus, in mice was formed through both monopodial and dipodial branching. However, our results suggest that the subsegmental bronchus in the human bronchial tree was generated only through monopodial branching. That is, the branching mode of the lobar bronchus and segmental bronchus in the murine bronchial tree coincided with that in the human lungs, whereas the branching mode of the subsegmental bronchus showed a difference between mice and humans in not only the branching mode but also the structural features. The branching pattern of the mouse lung is relatively stereotypic [2], but the branching tree in humans has more variations [6–10]. This variability in human lungs has been reported even during the embryonic period [4, 16]. Further investigations are needed to elucidate the differences in the mechanism of peripheral bronchi of mouse and human lungs.

Previous research indicated a remodeling mechanism called node retraction in the mouse kidney [17]. During node retraction, the PBr shortens after the CBr is generated. If this remodeling were to occur in the human lungs, then it would cause incorrect categorization of the branching mode. However, Watanabe et al. [3] observed morphogenesis of the mouse kidney until the eighth generation but did not report node retraction. Lindström et al. [17] attributed this difference to the insufficient culture period for node retraction in a previous study [3]. In the current study, the LSLB length did not decrease during our observation of morphogenesis of the LSLB until CS23 (the ninth generation from LSLB). Furthermore, the lengths of other analyzed bronchi did not show a decreasing trend (S2 Table). Therefore, our data did not suggest the occurrence of node retraction.

The current analysis suggests that the LSLB evidently bifurcated through dipodial branching. This was the only bronchus that was divided by dipodial branching in the present study. The mouse lungs do not have a bronchus that anatomically corresponds to superior division of the bronchus and LB because mice have only one lobe in the left lung. In comparison, in humans, the left lung consists of two lobes, and the left superior lobe is generally larger than the right superior lobe. Middle lobe syndrome and lingular syndrome are generally known as chronic inflammatory disorders that often occur in the right middle lobe and lingula. Therefore, from the viewpoint of clinical significance, the left superior bronchus seems to be a peculiar branch. The characteristic branching mode of the LSLB might reflect an anatomically unique structure that is specific to human lungs.

This study had some limitations. First, individual differences had an effect on the categorization of the branching mode because we used the minimum and maximum lengths for categorization in the current study. The right B5(TC) length had a wide range, which made it difficult to categorize the branching mode of the subsegmental bronchus. Second, we did not subdivide the branching modes into more groups; however, various branching modes have been reported. It would be possible to subdivide the branching modes into more than two modes by adding morphometric data such as angles or widths. Finally, some bronchi could not be determined accurately because of their immature structure at the segment level, which is why the subsegmental bronchus of the right B8 was not categorized as any branching mode.

## Conclusion

The present study analyzed the branching morphogenesis of the proximal bronchus by measuring the length. A morphometric analysis demonstrated that almost all proximal bronchi, except the LSLB, bifurcated with monopodial branching. Future analyses of parameters other than length, such as angles or widths, are needed to elucidate the branching morphogenesis.

## Supporting information

S1 Table Sample number grouped into a no-child group (NC) and a two-child group (TC) for each bifurcation. -, the bifurcation that was not grouped into NC or TC; NC, the bifurcation generating no-child branches; NC*, the bifurcation having probable B8 or B7+8; TC, the bifurcation generating two-child branches; LB, lingular bronchus; LILB, left inferior lobar bronchus; LSLB, left superior lobar bronchus; RILB, right inferior lobar bronchus; RMLB, right middle lobar bronchus; RSLB, right superior lobar bronchus; SDB, superior division bronchus.
(DOCX)

S2 Table The length of analyzed bronchi in each sample during CS16 and CS23. -, absent; n.d., not distinguished, Candidate bronchi were existing but could not be determined correctly; *, probable B8 or probable B7+8. LIL, left inferior lobe; LILB, left inferior lobar bronchus; LSL, left superior lobe; LSLB, left superior lobar bronchus; RIL, right inferior lobe; RILB, right inferior lobar bronchus; RML, right middle lobe; RMLB, right middle lobar bronchus; RSL, right superior lobe; RSLB, right superior lobar bronchus.
(DOCX)

S1 Fig Magnified figures provided in Fig 4 (The right superior lobe). (EPS)

S2 Fig Magnified figures provided in Fig 4 (The right middle lobe). (EPS)

S3 Fig Magnified figures provided in Fig 4 (The right inferior lobe). (EPS)

S4 Fig Magnified figures provided in Fig 4 [The left superior lobe (Superior division)]. (EPS)

S5 Fig Magnified figures provided in Fig 4 [The left superior lobe (Lingular division)]. (EPS)

S6 Fig Magnified figures provided in Fig 4 (The left inferior lobe). (EPS)
