# Supplementary material for: Bronchial tree of the human embryo: Categorization of the branching mode as monopodial and dipodial
Source: PLoS One. 2021 Jan 15;16(1):e0245558. doi: 10.1371/journal.pone.0245558 (PMC7810312; doi:10.1371/journal.pone.0245558)
Supplement: S1 Table — -, the bifurcation that was not grouped into NC or TC; NC, the bifurcation generating no-child branches; NC*, the bifurcation having probable B8 or B7+8; TC, the bifurcation generating two-child branches; LB, lingular bronchus; LILB, left inferior lobar bronchus; LSLB, left superior lobar bronchus; RILB, right inferior lobar bronchus; RMLB, right middle lobar bronchus; RSLB, right superior lobar bronchus; SDB, superior division bronchus. (DOCX) [file pone.0245558.s001.docx]

| CS | sample No | PBr (Lobar bronchus) | | | | | PBr (Segmental bronchus) | | | | | | | | | | | | | | | | | | | |
| --- | --- | --- | --- | --- | --- | --- | --- | --- | --- | --- | --- | --- | --- | --- | --- | --- | --- | --- | --- | --- | --- | --- | --- | --- | --- | --- |
|  |  | RSLB | RMLB | RILB | LSLB | LILB | B1 | B2 | B3 | B4 | B5 | B6 | B7 | B8 | B9 | B10 | SDB | B1+2 | B3 | LB | B4 | B5 | B6 | B7+8 | B9 | B10 |
| 15 | 15001 | - | - | - | - | - | - | - | - | - | - | - | - | - | - | - | - | - | - | - | - | - | - | - | - | - |
| 15 | 15025 | - | NC | NC | NC | NC | - | - | - | - | - | - | - | - | - | - | - | - | - | - | - | - | - | - | - | - |
| 15 | 15027 | - | - | - | - | - | - | - | - | - | - | - | - | - | - | - | - | - | - | - | - | - | - | - | - | - |
| 15 | 15041 | - | NC | NC | NC | NC | - | - | - | - | - | - | - | - | - | - | - | - | - | - | - | - | - | - | - | - |
| 15 | 15080 | - | - | - | - | - | - | - | - | - | - | - | - | - | - | - | - | - | - | - | - | - | - | - | - | - |
| 15 | 15103 | - | - | - | - | - | - | - | - | - | - | - | - | - | - | - | - | - | - | - | - | - | - | - | - | - |
| 15 | 15118 | - | - | - | - | - | - | - | - | - | - | - | - | - | - | - | - | - | - | - | - | - | - | - | - | - |
| 16 | 16009 | - | NC | NC | NC | NC | - | - | - | - | - | - | - | - | - | - | - | - | - | - | - | - | - | - | - | - |
| 16 | 16020 | NC | NC | NC | NC | NC | - | - | - | - | - | - | - | - | - | - | - | - | - | - | - | - | - | - | - | - |
| 16 | 16057 | NC | NC | NC | NC | NC | - | - | - | - | - | - | - | - | - | - | - | - | - | - | - | - | - | - | - | - |
| 16 | 16066 | NC | NC | NC | NC | NC | - | - | - | - | - | - | - | - | - | - | - | - | - | - | - | - | - | - | - | - |
| 16 | 16095 | NC | NC | NC | NC | NC | - | - | - | - | - | - | - | - | - | - | - | - | - | - | - | - | - | - | - | - |
| 16 | 16097 | NC | NC | NC | NC | NC | - | - | - | - | - | - | - | - | - | - | - | - | - | - | - | - | - | - | - | - |
| 16 | 16101 | NC | NC | NC | NC | NC | - | - | - | - | - | - | - | - | - | - | - | - | - | - | - | - | - | - | - | - |
| 17 | 17022 | - | TC | TC | TC | TC | - | NC | NC | NC | NC | NC | - | NC* | - | - | NC | - | - | NC | - | - | NC | NC* | - | - |
| 17 | 17052 | TC | TC | TC | TC | NC | NC | NC | NC | NC | NC | NC | NC | NC* | - | - | NC | - | - | NC | - | - | - | NC* | - | - |
| 17 | 17055 | TC | TC | TC | - | TC | NC | NC | NC | NC | NC | NC | NC | NC | - | - | TC | NC | NC | NC | - | - | NC | TC | - | - |
| 17 | 17057 | TC | TC | TC | - | TC | NC | NC | NC | NC | NC | NC | - | NC | NC | NC | TC | NC | NC | NC | - | - | NC | NC | NC | NC |
| 17 | 17082 | - | NC | TC | TC | NC | - | NC | NC | - | - | NC | - | NC* | - | - | NC | - | - | NC | - | - | - | - | - | - |
| 17 | 17100 | NC | NC | TC | TC | TC | - | - | - | - | - | NC | - | NC* | - | - | NC | - | - | NC | - | - | NC | NC* | - | - |
| 17 | 17118 | TC | TC | TC | TC | TC | NC | NC | NC | NC | NC | NC | - | NC* | - | - | NC | - | - | NC | - | - | NC | NC* | - | - |
| 18 | 18005 | - | TC | - | - | - | NC | NC | TC | NC | TC | TC | - | TC | TC | TC | - | TC | - | TC | NC | TC | TC | NC | NC | TC |
| 18 | 18010 | - | - | - | - | - | NC | TC | TC | - | - | TC | TC | TC | NC | NC | TC | TC | NC | TC | NC | NC | TC | TC | NC | NC |
| 18 | 18031 | - | - | - | - | - | TC | TC | TC | - | - | TC | - | TC | NC | TC | - | - | TC | TC | NC | NC | TC | TC | TC | NC |
| 18 | 18041 | - | TC | TC | TC | TC | NC | NC | NC | NC | - | NC | - | - | - | - | NC | - | - | NC | - | - | NC | NC* | - | - |
| 18 | 18071 | - | TC | - | - | - | TC | NC | - | NC | - | - | TC | TC | NC | - | - | - | - | TC | NC | NC | - | TC | TC | TC |
| 18 | 18083 | - | TC | TC | TC | TC | - | NC | NC | NC | NC | NC | NC | NC* | - | - | NC | - | - | NC | - | - | NC | NC* | - | - |
| 18 | 18105 | - | TC | - | - | TC | NC | NC | NC | NC | TC | NC | TC | NC* | - | - | TC | NC | NC | NC | - | - | NC | NC | NC | NC |
| 19 | 19006 | - | - | - | - | - | NC | TC | TC | TC | TC | - | TC | TC | NC | NC | - | TC | TC | TC | NC | TC | - | - | - | - |
| 19 | 19020 | - | - | - | - | - | - | TC | TC | - | - | - | TC | TC | TC | - | - | TC | TC | - | TC | TC | TC | - | TC | - |
| 19 | 19023 | - | - | - | - | - | TC | TC | - | TC | - | - | TC | - | TC | - | TC | - | NC | - | TC | NC | - | - | TC | - |
| 19 | 19040 | - | - | - | - | - | NC | NC | TC | TC | TC | TC | TC | TC | NC | NC | TC | NC | NC | TC | NC | NC | TC | TC | NC | NC |
| 19 | 19049 | - | - | - | - | - | - | - | - | - | TC | - | TC | TC | - | - | - | - | - | - | - | - | - | - | TC | TC |
| 19 | 19054 | - | - | - | - | - | - | - | TC | TC | TC | TC | NC | - | TC | TC | NC | - | - | - | - | - | - | TC | TC | - |
| 19 | 19057 | - | TC | - | - | - | TC | TC | NC | NC | TC | - | TC | TC | NC | TC | TC | - | NC | TC | NC | NC | TC | - | NC | NC |
