# Supplementary material for: Bronchial tree of the human embryo: Categorization of the branching mode as monopodial and dipodial
Source: PLoS One. 2021 Jan 15;16(1):e0245558. doi: 10.1371/journal.pone.0245558 (PMC7810312; doi:10.1371/journal.pone.0245558)
Supplement: S2 Table — -, absent; n.d., not distinguished, Candidate bronchi were existing but could not be determined correctly; *, probable B8 or probable B7+8. LIL, left inferior lobe; LILB, left inferior lobar bronchus; LSL, left superior lobe; LSLB, left superior lobar bronchus; RIL, right inferior lobe; RILB, right inferior lobar bronchus; RML, right middle lobe; RMLB, right middle lobar bronchus; RSL, right superior lobe; RSLB, right superior lobar bronchus. (DOCX) [file pone.0245558.s002.docx]

| CS | sample No | Right lobe | | | | | | | | | | | | | | | | Left lobe | | | | | | | | | | | |
| --- | --- | --- | --- | --- | --- | --- | --- | --- | --- | --- | --- | --- | --- | --- | --- | --- | --- | --- | --- | --- | --- | --- | --- | --- | --- | --- | --- | --- | --- |
|  |  | **Branch# (RSL)** | **RSLB** | **B1** | **B2** | **B3** | **Branch# (RML)** | **RMLB** | **B4** | **B5** | **Branch# (RIL)** | **RILB** | **B6** | **B7** | **B8** | **B9** | **B10** | **Branch# (LSL)** | **LSLB** | **B1+2** | **B3** | **B4** | **B5** | **Branch# (LIL)** | **LILB** | **B6** | **B7+8** | **B9** | **B10** |
| 16 | 16009 | 0 | - | - | - | - | 1 | 131.1 | - | - | 1 | 350.6 | - | - | - | - | - | 1 | 84.3 | - | - | - | - | 1 | 284.7 | - | - | - | - |
| 16 | 16020 | 1 | 117.0 | - | - | - | 1 | 152.0 | - | - | 1 | 341.6 | - | - | - | - | - | 1 | 92.2 | - | - | - | - | 1 | 312.1 | - | - | - | - |
| 16 | 16057 | 1 | 135.2 | - | - | - | 1 | 131.9 | - | - | 1 | 246.7 | - | - | - | - | - | 1 | 138.8 | - | - | - | - | 1 | 288.2 | - | - | - | - |
| 16 | 16066 | 1 | 167.9 | - | - | - | 1 | 145.7 | - | - | 1 | 284.9 | - | - | - | - | - | 1 | 69.3 | - | - | - | - | 1 | 264.0 | - | - | - | - |
| 16 | 16095 | 1 | 167.3 | - | - | - | 1 | 148.7 | - | - | 1 | 291.1 | - | - | - | - | - | 1 | 126.2 | - | - | - | - | 1 | 297.6 | - | - | - | - |
| 16 | 16097 | 1 | 247.7 | - | - | - | 1 | 259.8 | - | - | 1 | 571.6 | - | - | - | - | - | 1 | 126.7 | - | - | - | - | 1 | 484.3 | - | - | - | - |
| 16 | 16101 | 1 | 132.5 | - | - | - | 1 | 74.9 | - | - | 1 | 284.1 | - | - | - | - | - | 1 | 77.9 | - | - | - | - | 1 | 316.1 | - | - | - | - |
| 17 | 17022 | 3 | 315.0 | - | 187.2 | 217.3 | 3 | 331.0 | 199.6 | 174.6 | 5 | 202.7 | 313.3 | - | 245.3* | - | - | 3 | 226.2 | - | - | - | - | 5 | 202.6 | 271.4 | 229.8* | - | - |
| 17 | 17052 | 4 | 271.9 | 176.3 | 189.0 | 322.3 | 3 | 300.3 | 251.7 | 219.1 | 7 | 92.1 | 285.6 | 333.0 | 252.4* | - | - | 3 | 140.6 | - | - | - | - | 3 | 337.8 | - | 217.8* | - | - |
| 17 | 17055 | 5 | 210.0 | 192.6 | 200.7 | 258.1 | 3 | 260.7 | 139.1 | 239.0 | 7 | 173.4 | 271.1 | 240.4 | 218.3 | - | - | 5 | 147.5 | 196.5 | 200.7 | - | - | 7 | 207.4 | 266.7 | 112.1* | - | - |
| 17 | 17057 | 5 | 228.8 | 226.7 | 199.4 | 224.3 | 3 | 247.6 | 198.2 | 264.8 | 7 | 152.8 | 250.5 | - | 174.9 | 184.6 | 265.8 | 5 | 200.3 | 190.2 | 190.0 | - | - | 7 | 149.5 | 273.5 | 218.8 | 210.3 | 334.6 |
| 17 | 17082 | 3 | 271.6 | - | 113.4 | 247.9 | 1 | 451.1 | - | - | 5 | 177.5 | 221.1 | - | 183.1* | - | - | 3 | 167.4 | - | - | - | - | 3 | 414.6 | - | - | - | - |
| 17 | 17100 | 1 | 381.6 | - | - | - | 1 | 412.4 | - | - | 5 | 133.6 | 247.0 | - | 192.7* | - | - | 3 | 198.0 | - | - | - | - | 5 | 211.7 | 243.9 | 213.6* | - | - |
| 17 | 17118 | 4 | 229.1 | 187.4 | 191.8 | 308.7 | 3 | 205.9 | 245.5 | 183.0 | 5 | 117.4 | 271.6 | - | 246.3* | - | - | 3 | 201.5 | - | - | - | - | 5 | 147.7 | 205.3 | 252.7* | - | - |
| 18 | 18005 | 7 | 313.0 | 348.5 | 362.5 | 288.7 | 5 | 367.3 | 573.6 | 195.4 | 15 | 162.5 | 299.4 | - | 280.5 | 245.2 | 128.9 | 15 | 175.6 | 230.6 | 56.6 | 362.9 | 188.4 | 11 | 297.9 | 243.4 | 508.5 | 362.5 | 284.8 |
| 18 | 18010 | 9 | 315.3 | 372.7 | 166.2 | 160.5 | 7 | 328.5 | n.d. | n.d. | 15 | 184.9 | 182.4 | 323.0 | 186.4 | 504.0 | 584.1 | 9 | 237.9 | 53.9 | 135.7 | 239.5 | 282.2 | 11 | 299.6 | 227.5 | 197.0 | 404.8 | 469.4 |
| 18 | 18031 | 11 | 154.7 | 199.6 | 188.0 | 206.5 | 9 | 262.3 | n.d. | n.d. | 13 | 121.4 | 182.7 | - | 213.1 | 340.9 | 163.4 | 15 | 208.0 | 70.8 | 255.4 | 147.9 | 177.1 | 13 | 239.9 | 258.3 | 210.8 | 264.7 | 499.6 |
| 18 | 18041 | 5 | 305.7 | 216.0 | 219.9 | 270.2 | 3 | 241.6 | 272.8 | n.d. | 3 | 102.4 | 294.9 | - | - | - | - | 3 | 196.1 | - | - | - | - | 5 | 172.0 | 339.6 | 275.0 | - | - |
| 18 | 18071 | 10 | 270.8 | 213.7 | 406.4 | 130.0 | 7 | 319.7 | 436.0 | 153.5 | 21 | 43.2 | 179.4 | 201.4 | 190.6 | 400.8 | 176.3 | 15 | 220.7 | 78.7 | 106.3 | 211.9 | 216.7 | 17 | 179.2 | 219.9 | 208.2 | 169.1 | 370.8 |
| 18 | 18083 | 3 | 318.1 | - | 259.4 | 320.8 | 3 | 287.3 | 335.3 | 276.4 | 7 | 81.4 | 396.5 | 328.6 | 306.6* | - | - | 3 | 221.3 | - | - | - | - | 5 | 213.0 | 380.8 | 270.5 | - | - |
| 18 | 18105 | 5 | 282.2 | 270.4 | 273.2 | 385.2 | 5 | 290.8 | 243.3 | 151.8 | 9 | 157.9 | 432.3 | 176.6 | 288.1* | - | - | 5 | 220.0 | 255.9 | 254.9 | - | - | 7 | 258.9 | 464.2 | 327.6 | 230.3 | 301.5 |
| 19 | 19006 | 11 | 374.3 | 365.1 | 246.3 | 262.2 | 7 | 300.0 | 220.5 | 450.3 | 21 | 169.1 | 237.2 | 283.4 | 246.7 | 267.1 | 342.5 | 13 | 203.0 | 65.0 | 200.5 | 308.8 | 159.3 | 17 | 151.4 | 197.0 | n.d. | n.d. | n.d. |
| 19 | 19020 | 15 | 320.9 | 286.3 | 104.0 | 191.6 | 9 | 255.3 | n.d. | n.d. | 27 | 167.4 | 242.0 | 303.3 | 206.9 | 247.7 | 221.9 | 19 | 264.4 | 186.1 | 185.1 | 175.4 | 210.3 | 22 | 258.0 | 308.5 | 104.0 | 196.8 | 320.5 |
| 19 | 19023 | 19 | 339.4 | 318.2 | 291.3 | 217.4 | 9 | 569.8 | 161.9 | 18.4 | 31 | 128.3 | 288.4 | 433.3 | 262.2 | 281.9 | 87.4 | 15 | 294.4 | 40.9 | 460.7 | 198.3 | 413.1 | 31 | 179.0 | 304.9 | 365.6 | 171.7 | 267.9 |
| 19 | 19040 | 7 | 234.5 | 433.0 | 406.8 | 284.5 | 7 | 296.4 | 198.1 | 306.9 | 15 | 195.1 | 188.9 | 338.0 | 198.0 | 418.9 | 462.8 | 7 | 283.9 | 277.7 | 334.0 | 207.0 | 228.1 | 11 | 243.6 | 261.1 | 253.0 | 383.3 | 445.3 |
| 19 | 19049 | 23 | 254.2 | 264.7 | 267.4 | 268.8 | 11 | 343.4 | 204.4 | 209.8 | 29 | 98.5 | 293.1 | 429.7 | 310.1 | 209.6 | 173.4 | 21 | 254.7 | n.d. | n.d. | n.d. | n.d. | 28 | 206.9 | 351.5 | 323.9 | 128.2 | 310.9 |
| 19 | 19054 | 15 | 324.5 | 189.2 | 198.0 | 147.6 | 11 | 377.1 | 352.3 | 36.8 | 21 | 80.1 | 290.6 | 621.2 | 320.7 | 273.0 | 313.1 | 21 | 224.7 | 133.7 | 178.1 | n.d. | n.d. | 23 | 220.9 | 315.2 | 203.7 | 214.8 | 226.5 |
| 19 | 19057 | 11 | 229.6 | 211.9 | 239.3 | 186.3 | 5 | 317.4 | 359.1 | 200.9 | 19 | 100.3 | 187.3 | 257.1 | 224.2 | 430.5 | 103.6 | 11 | 205.1 | 97.1 | 414.0 | 323.5 | 361.9 | 15 | 223.9 | 295.7 | 158.5 | 353.6 | 411.3 |
| 20 | 20005 | 39 | 331.6 | 134.0 | 247.1 | 215.6 | 17 | 468.3 | 214.3 | 369.6 | 61 | 109.1 | 135.1 | 347.0 | 322.1 | 145.7 | 306.9 | 41 | 199.8 | 285.2 | 285.9 | 140.0 | 462.9 | 47 | 172.6 | 296.8 | 281.3 | 162.3 | 444.0 |
| 20 | 20035 | 23 | 351.2 | n.d. | n.d. | n.d. | 13 | 289.2 | 170.9 | 144.2 | 35 | 148.9 | 97.4 | 553.7 | 426.6 | 302.2 | 108.2 | 23 | 159.6 | 50.9 | 383.4 | 115.4 | 316.5 | 25 | 161.0 | 212.9 | 164.7 | 653.4 | 199.5 |
| 20 | 20053 | 25 | 213.6 | 95.8 | 344.5 | 264.4 | 11 | 323.9 | 302.1 | 258.5 | 39 | 60.2 | 204.5 | 227.5 | 372.5 | 248.5 | 109.0 | 24 | 182.7 | 157.6 | 213.2 | n.d. | n.d. | 39 | 211.2 | 331.7 | n.d. | n.d. | n.d. |
| 20 | 20054 | 35 | 324.9 | 199.9 | 221.7 | 227.0 | 20 | 331.4 | 287.5 | 135.3 | 66 | 78.8 | 220.9 | 226.7 | 175.5 | 251.3 | 143.4 | 39 | 230.1 | n.d. | n.d. | n.d. | n.d. | 59 | 205.0 | 182.1 | 272.8 | 278.7 | 338.5 |
| 21 | 21039 | 73 | 322.3 | 359.8 | 318.2 | 91.1 | 41 | 422.1 | 195.7 | 170.6 | 108 | 162.5 | 200.5 | 241.9 | 197.3 | 341.8 | 291.1 | 75 | 347.6 | 198.1 | 107.0 | n.d. | n.d. | 95 | 172.2 | 371.3 | 143.0 | 377.8 | 267.7 |
| 21 | 21056 | 41 | 349.6 | - | 108.6 | 184.4 | 21 | 352.4 | 172.0 | 139.8 | 65 | 25.5 | 258.7 | 163.1 | n.d. | n.d. | n.d. | 49 | 206.8 | 115.7 | 130.2 | 222.1 | 410.2 | 56 | 169.8 | 367.0 | 239.2 | 261.9 | 192.5 |
| 21 | 21079 | 47 | 404.0 | - | 221.7 | 264.7 | 35 | 371.2 | 237.5 | 154.3 | 75 | 74.3 | 197.6 | 364.2 | 223.9 | 57.4 | 225.4 | 39 | 323.8 | 394.5 | 136.7 | 271.8 | 163.6 | 83 | 194.1 | 266.1 | 240.3 | 138.9 | 256.3 |
| 21 | 21120 | 83 | 261.8 | 287.0 | 273.9 | 219.7 | 45 | 320.7 | 204.0 | 260.0 | 123 | 132.7 | 169.8 | 484.0 | 169.8 | 192.2 | 265.0 | 97 | 269.1 | 179.6 | 254.0 | 260.7 | 243.4 | 138 | 241.3 | 287.3 | 174.9 | 229.1 | 219.0 |
| 22 | 22007 | 63 | 102.6 | 278.1 | 156.7 | 198.7 | 47 | 246.2 | 105.2 | 114.5 | 85 | 178.2 | 153.2 | - | 112.8 | 199.8 | 188.9 | 71 | 202.0 | n.d. | n.d. | n.d. | n.d. | 87 | 526.3 | 203.5 | 161.5 | 239.7 | 331.9 |
| 22 | 22033 | 95 | 376.2 | 301.0 | 217.7 | 146.8 | 55 | 438.3 | 274.5 | 203.7 | 135 | 97.5 | 241.1 | 669.3 | 291.5 | 606.8 | 303.4 | 109 | 277.9 | n.d. | n.d. | n.d. | n.d. | 142 | 320.1 | 219.9 | 275.8 | 210.0 | 234.4 |
| 22 | 22045 | 103 | 266.1 | 191.0 | 290.4 | 197.0 | 46 | 348.6 | 334.2 | 123.1 | 159 | 56.4 | 298.4 | 409.8 | 196.9 | 359.0 | 181.6 | 119 | 262.5 | 78.2 | 238.1 | 276.6 | 245.4 | 169 | 184.0 | 289.0 | 324.7 | 368.1 | 202.9 |
| 23 | 23008 | 134 | 369.1 | 442.7 | 288.4 | 216.2 | 74 | 419.1 | 315.1 | 434.8 | 209 | 110.8 | 309.9 | 350.7 | 284.8 | 321.7 | 338.1 | 152 | 262.5 | n.d. | n.d. | n.d. | n.d. | 183 | 205.0 | 221.4 | 259.1 | n.d. | 177.6 |
| 23 | 23053 | 159 | 393.6 | 402.1 | 333.1 | 367.5 | 97 | 411.3 | 381.6 | 148.6 | 279 | 94.1 | 279.5 | 387.5 | 323.9 | 283.2 | 346.8 | 194 | 314.9 | 240.8 | 387.5 | 273.3 | 263.8 | 261 | 248.5 | 427.4 | 371.1 | 446.0 | 272.5 |
